# Culture in Physical Activity: The Contribution of Basic Psychological Needs and Goal Orientation

**DOI:** 10.3390/ijerph192416691

**Published:** 2022-12-12

**Authors:** Duygu Gurleyik, Celia K. Naivar Sen, Jennifer L. Etnier, Ibrahim H. Acar

**Affiliations:** 1 Department of Psychology, Özyeğin University, Çekmeköy, Istanbul 34794, Turkey; 2 Department of Kinesiology, University of North Carolina at Greensboro, Greensboro, NC 27412, USA

**Keywords:** physical activity, culture, autonomy, competence, relatedness, ego, task

## Abstract

Numerous variables affect motivation in physical activity (PA) with culture being an understudied variable. Self-determination theory’s basic psychological needs (BPN) includes a combination of autonomy, competence, and relatedness in PA; however, cultural definitions pit autonomy and relatedness against each other. Thus, this study aims to investigate the moderating role of culture on relationships between BPN, goal orientations (ego, task) for PA, and PA behavior. A survey was implemented to 168 participants (109 females, 59 males; 92 Turks, 76 Americans) investigating students’ self-construal type, their basic psychological needs in exercise (BPNES), PA levels (Godin Leisure-Time Exercise Questionnaire/GLTEQ), and goal orientation types (Task and Ego Orientation in Sport Questionnaire/TEOSQ). Turks (*n* = 92) and Americans (*n* = 76) demonstrated distinct cultural differences in terms of the study variables. American students were more autonomous, task-oriented, and physically active than Turkish students. Results from the multi-group path analysis showed that there was a moderating role of culture between predictors (i.e., BPN Autonomy, BPN Relatedness, BPN Competence, Ego Orientation, and Task Orientation) and Physical Activity. Such that, the paths from predictors (i.e., BPNT Autonomy, BPNT Relatedness, BPNT Competence, Ego Orientation, and Task Orientation) to PA was not significant in Turkish cultural context. Results suggest that culturally tailored approaches to PA interventions are critical in supporting motivation for physical activity and further research is needed to explore different culturally relevant motivational drivers for PA among adults.

## 1. Introduction

Given the global pervasiveness of public health campaigns, people readily acknowledge the health benefits of physical activity (PA) and the risks of inactivity. Unfortunately, having this knowledge alone is not sufficient to motivate behavior change [1]. While the recommended guideline of 150 min weekly of PA (e.g., brisk walking) is widely publicized, many people have difficulties meeting this recommendation. According to recent WHO reports on physical activity, globally, one out of four adults do not meet recommended levels of PA [2].

Taking into account the low PA levels in adults, new research is needed to understand the epidemic of inactivity. As lack of adequate PA is a global problem, one understudied element potentially affecting PA is culture [3]. Thus, the aim of this work is to investigate how culture may relate to PA within two major PA frameworks discussed shortly; (1) self-determination theory’s (SDT) basic needs of competence, autonomy, and relatedness [4,5] and (2) goal orientation’s drives of task mastery and competition [6,7].

### 1.1. Culture Theories and the Role of Autonomy and Relatedness

Prior to discussing culture’s role in PA and specifically within SDT and goal orientation frameworks, it is useful to understand cultural theories. One of the foremost conceptualisms of culture is the designation of individualist and collectivist cultures [8,9] and the corresponding independent and interdependent self-construals within each culture [10]. Individualistic nations are described as egocentric societies, giving importance to individual goals and autonomy. In individualistic cultures such as those in North America and Western Europe [11,12,13], the independent self-construal dominates opting for autonomy, self-reliance, and the expression of unique inner attributes rather than connectedness. For the independent construal, achievement is considered a personal success.

Contrastingly, Asia, Africa, Latin America, the Middle East, and Southern Europe are examples of collectivistic cultures [11,12,13] valuing communality, group goals, and relatedness [13,14]. Here, the interdependent construal dominates with conceptions of the self, based upon the fundamental relatedness of individuals within a group, with success measured by shared goals and group cohesiveness.

Within these traditional prevailing models, autonomy and relatedness are seen as competing rather than co-existing entities. To resolve the inherent conflict between autonomy and relatedness when considering independent and interdependent self-construals, Kagitcibasi [13,15] proposed the autonomous-related self, where both autonomy and relatedness are considered as distinct entities rather than competing constructs. She explains that, in the dichotomization of cultures, autonomy is often confused with being an independent agent—one who is separate from others. In contrast, relatedness is often confused with interdependence. However, a person can autonomously decide to depend on someone else. Therefore, autonomy does not necessarily refer to independence, and people can simultaneously experience the basic needs of autonomy and relatedness. In this sense, autonomy is not experienced at the expense of relatedness and vice versa.

### 1.2. Self-Determination Theory: Competence, Autonomy, and Relatedness

When considering culture and its association with PA, the concept of autonomy and relatedness coexisting rather than competing aligns with SDT and its sub-theory, basic psychological needs theory (BPNT). BPNT explains that for all individuals, competence, autonomy, and relatedness are basic needs that must be experienced simultaneously for a person to be motivated and function optimally for a given task [4]. The first need, competence, encompasses being qualified in a specific skill. In line with this need, people must feel a sense of mastery over a task and will choose activities they think they are good at doing (e.g., if they view themselves as competent at running, they may choose to be active through jogging). The second need, autonomy, involves the ability to make decisions without outside control. Individuals with higher autonomy perceive their PA behaviors to be self-regulated, intentional, and volitional. The third and final need is relatedness. Relatedness is a feeling of belongingness in a group. People need to perceive the connection within the members of the PA group to have a sustainable motivation [4,16]. Thus, people will function effectively and become motivated when autonomy, relatedness, and competence are fulfilled [16,17].

In addition to these three needs, BPNT also emphasizes the importance of the environment [5,18]. The environment, of which culture is one aspect, should provide a milieu enabling the three basic needs’ fulfillment. Cultural values are known to influence a myriad of behaviors such as consumer behavior, food choices, health beliefs, etc. [19,20,21], and PA behavior may also be influenced by the culture in which it is situated. Thus, for needs to be met relative to PA, culture should foster an environment in which these three basic needs may be experienced simultaneously when electing to participate in PA behaviors [22,23].

The multicultural nature of PA and exercise is often disregarded, the majority of the research was conducted was with an easily convenient and available sample (white male participants form individualistic countries). Therefore, there have been concerns about proper and ethical psychological findings to culturally diverse clients. Thus culturally competence research in sport an exercise psychology was scarce and there have been calls for conducting such research [3]. Even though cultural diversity is rarely investigated in the field of sport and exercise psychology [11,12,17,24,25], these cultural differences (i.e., independent, interdependent), which are commonly investigated in social psychology, may foster different environments fulfilling different levels of BPNT PA needs. As stated previously, Eastern cultures prioritize relatedness while Western cultures prioritize autonomy. This cultural dichotomization of setting relatedness against autonomy is in direct opposition to BPNT where autonomy, relatedness, and competence must coexist. Therefore, considering culture and how levels of relatedness and autonomy in the self-interact with BPNT could shed light on the global PA problem.

### 1.3. Goal Orientation and Culture

In addition to the previously discussed role culture has played in BPNT, one may also see cultural differences in goal orientation. Nicholls’s (1989) [26] goal orientation perspective includes two different goal orientations: task orientation and ego orientation. People who are raised in societies that promote relatedness and collectivism are expected to be more task-oriented, an orientation that focuses on working hard to learn and improve a particular skill and desire to become an expert at that particular skill with the goal of personal development [12]. On the other hand, people who are raised in societies that promote autonomy and foster individualism are expected to be more ego-oriented with the main goal of out-performing their opponents [11,27,28]. However, as indicated by Gill and Williams [29] and as could be seen in prevailing cultural models, this dichotomy oversimplifies these concepts, as there are no strict boundaries that separate individualism/ collectivism and ego-orientation/task-orientation. Thus, viewing autonomy and relatedness, as well, as task and ego orientation as distinct constructs rather than competing entities may provide a more comprehensive picture of PA in various cultures.

### 1.4. Current Study

In the current study, culture, provided a bridge to consider how BPNT and goal orientations are related to PA behavior in Turkey and the United States of America (USA). Here, the two constructs—autonomy and relatedness—were treated as distinct entities rather than two poles of a continuum, allowing both to coexist in the two cultures [15]. As Turkey is generally categorized as interdependent and the USA as an independent nation [13], the following differences were expected between the two cultures for self-construals: Based on literature, Turks were expected to report lower levels of autonomy and higher levels of relatedness compared to Americans [11,14]. Even though differences between cultures were expected, the overall self-autonomy and relatedness scores in both cultures were predicted to be at least moderate as both constructs are basic needs for humans [13,15].

As for BPNT in PA, due to influences of culture in the environment (i.e., cultural fostering of relatedness for Turks and autonomy for Americans [11,14]), it was hypothesized that Turks would have higher levels of relatedness in PA settings, while Americans would have higher levels of autonomy in PA settings. Again, while differences were expected based on the environment fostered by cultural priorities, both groups should have basic levels of each construct. Additionally, for the third BPN, both groups were expected to have similar levels of need for competence in PA settings as this need is not dichotomized in cultural research [11,14]. For goal orientation, as ego orientation was associated with independent cultures [11,27,28], Turks were expected to be more task-oriented and less ego-oriented in their PA participation in comparison to Americans.

When considering how basic psychological needs are related to PA, it was predicted that autonomy, relatedness, and competence would be positively related to PA levels [4]. Furthermore, as both goal motivation constructs are positively associated with higher PA levels [26], similar findings were expected. Finally, it was expected that viewing the model in a way to see the direct effect of nationality, as well as the interaction of nationality and BPNT/goal orientation on PA levels, would increase the strength of the model. In this model, it was expected that country, as a proxy for culture, would moderate the relationship between BPNT autonomy, BPNT relatedness, and both goal orientations on PA such that Americans would have stronger associations between both autonomy and ego orientation on PA. It is thus expected that the higher levels of autonomy and ego orientations in Americans would be associated with higher PA levels. Turks are expected to have stronger associations between both relatedness and task orientation, and thus expected to have higher PA levels associated with this route.

## 2. Materials and Methods

### 2.1. Participants

In the current cross-sectionally designed study, there were 168 participants in total (109 females, 59 males). Participants were between 18 and 50 years of age (M = 22.39, SD = 4.19). Turkish participants were recruited from Koç University in Istanbul, Turkey (n = 92, 62 females, 30 males: 100% Caucasian). American participants were recruited from Greensboro, North Carolina, United States (University of North Carolina at Greensboro, UNCG) (n = 76; 47 females, 29 males; 69.7% Caucasian, 23.7% African American, 2.6% Asian, and 1.3% of each Native American, Hispanic, and other). All participants were recruited via flyers and class announcements done by psychology professors. Participation was on a voluntary basis in which they earned for one extra credit for the course the student was enrolled in. As nationality was an important variable, of the original 171 participants, three participants who were not born in the current country of residence were excluded from the study. There was no sex (*χ*^2^ (1) = 0.562, *p* = 0.56) and age difference (*t* (166) = 1.003, *p* = 0.317) between American and Turkish participants. 

### 2.2. Procedure

The study was approved by the Institutional Review Board at UNCG with a code number 12-0004 on 7 February 2012. We utilized nonprobability convenience sampling to reach out to students at researchers’ institutions in the U.S. and Turkey. Announcement flyers were available on boards throughout both universities. Recruitment announcements were also made verbally and in writing in various psychology elective classes. These electives were open to many students from different majors (e.g., Engineering, architecture, business administration, etc.) The announcement was about the nature and aims of the study, and that their participation was anonymous and voluntary. Participants completed the pen and paper questionnaire anonymously, and forms were collected by research assistants. As the language of instruction in both universities was English, English questionnaires were used at both institutions. Prior to survey administration, informed consent was obtained. Participants who were taking psychology electives were able to receive one credit for the class upon request. All collected data and entered into SPSS. Although questionnaires had no identifying information other than age and nationality, they were kept in a locked cabinet with digital data stored on password-protected computer. Only the authors had access to the data. The survey was in person and took approximately 15 min to complete.

### 2.3. Measures

The questionnaire, completed via pen and paper, consisted of five sections: demographic information, the Self-in-Family Scale, Basic Psychological Needs in Exercise Scale (BPNES), the Task and Ego Orientation in Sport Questionnaire (TEOSQ), and the Godin Leisure-Time Exercise Questionnaire (GLTEQ). The demographic information consisted of participants’ gender, age, nationality, and ethnicity.

#### 2.3.1. Self-in-Family Scale (Self-Construals)

Participants completed a questionnaire to determine their self-construals [15]. The survey was designed to measure the construals within the family and consisted of eighteen items with two subscales: nine measuring the autonomous self (e.g., “I feel independent of my family”), nine measuring the related self (e.g., “Feeling very close to my family is a good thing”). A 5-point Likert scale was used for the survey (1 = strongly disagree to 5 = strongly agree). The reliability of both autonomous- and related-self scales has been previously established (α = 0.84) [15]. In the current study, item 15 was dropped due to low reliability for autonomy. Thus, autonomy’s reliability was 0.77 and relatedness was 0.88 in this sample.

#### 2.3.2. Basic Psychological Needs in Exercise Scale (BPNES)

The psychological needs of SDT were assessed using the basic psychological needs in exercise scale (BPNES). This scale consisted of three different scales that measure the basic needs of autonomy, relatedness, and competence in an exercise setting. Of the eleven questions, four measured autonomy (e.g., “I feel the way I exercise is a way that I want to”), four measured competency (e.g., “I feel exercise is an activity which I do very well”), and the remaining three measured relatedness (e.g., “My relationships with the people I exercise with are close”). The reliability of the autonomy, competence and relatedness scales were 0.75, 0.80, and 0.86, respectively [30]; while in the present sample, the reliability scores were 0.78, 0.82, and 0.86, respectively.

#### 2.3.3. Task and Ego Orientation Scale Questionnaire (TEOSQ)

Both task and ego orientation in PA was measured using the TEOSQ. The survey consisted of thirteen items. Seven items measured task orientation (e.g., “I feel the most successful in sport when something I learn makes me want to go practice more”), and the remaining six questions measured ego orientation (e.g., “I feel the most successful in sport when I can do better than my friends”) on 5-point Likert scales (1 = strongly disagree to 5 = strongly agree). In previous research, the task orientation scale reliability ranged from 0.71 to 0.86 and the reliability of the ego orientation scale ranged from 0.79 to 0.90 [27]. The current reliability was 0.87 for ego and 0.81 for task orientation. The TEOSQ questionnaire was originally designed with each item including the word “sport”. In the current study, the word “sport” was replaced by “physical activity.” PA was defined at the beginning of the questionnaire as any structured or unstructured activity, including school-based physical education, recreational activities, dance, going to the gym, college club activities such as volleyball, basketball, soccer, trekking, snowboarding, tennis, and kayaking, and active transport such as walking and biking.

#### 2.3.4. Godin Leisure Time Exercise Questionnaire (GLTEQ)

PA was assessed using the GLTEQ. This self-report survey asked participants the amount and type of PA that they engaged in for more than fifteen minutes during a seven-day period. The total score for PA was calculated by multiplying weekly rates of strenuous, moderate, and light activities by nine, five, and three, respectively and then summing the scores. The 2-week test–retest reliability of the measures of total leisure activity have been found to be 0.74, with higher scores reflecting high levels of physical activity [31].

### 2.4. Analysis

There was no missing data in the current study. We examined the skewness and kurtosis of the predictor variables to investigate normality assumptions of the distribution for each variable (criteria for skewness and kurtosis was ±2, Gravetter and Wallnau 2014; Trochim and Donnelly 2006). Skewness values ranged from −0.957 to −0.024 and kurtosis values ranged from −0.319 to 1.11. None of the variables were out of acceptable range for non-normality; therefore, no transformation was applied. We ran a multicollinearity test on the predictor variables to examine whether they were highly correlated with one another in predicting the dependent variable [32]. The results showed that variance inflation factors ranged from 1.02 to 2.46 and tolerance values ranged from 0.40 to 0.95, indicating there was no multicollinearity issue among our focal variables [33]. Path analysis was run using the *Mplus 8.4* [34]. Path analysis is a special case of structural equation model that uses only observed variables rather than latent variables in statistical testing [35]. We used each subscale of the target constructs to test their unique contribution to physical activity in both cultural groups. Further, multi-group analysis was employed to test the moderating role of cultural context on the association between predictors (i.e., BPNT Autonomy, BPNT Relatedness, BPNT Competence, Ego Orientation, and Task Orientation) and PA. Multi-group analysis in a path modeling context could be used to test the moderating role of the categorical variable [35], referring to two cultural groups in the current study. A post hoc power analysis using regression analysis was employed to examine whether there was enough power to detect effects [36,37]. The power analyses revealed that at α = 0.05 and given an effect size of 0.21 (the smallest effect size for regression models in the current study), statistical power of 0.87 was gathered with n = 72, the smallest n for given groups.

## 3. Results

### 3.1. Preliminary Results

Before accepting nationality as a proxy for culture, the differences in self-autonomy and relatedness were analyzed. As expected, Americans (M = 3.70, SD = 0.58) were significantly higher in the autonomy self-construal than Turks (M = 3.43, SD = 0.68), t(166) = 2.79, *p* ≤ 0.01. On the other hand, Turks (M = 4.20, SD = 0.66) were significantly higher in the relatedness self-construal than Americans (M = 3.79, SD = 0.81), t(166) = 3.62, *p* < 0.000 (see Table 1). Both groups were at least moderate in each construct showcasing that while differences exist, the constructs are not at odds with each other. From this point on in analyses, Turks and Americans are considered as two distinct groups.

Results from bivariate correlation (Pearson) analysis showed that physical activity was related to BPNT autonomy (*r* = 0.23, 0.31) and BPNT competence (*r* = 0.51, 0.32) for American and Turkish samples, respectively. Physical activity was related to ego orientation (*r* = 0.23) and task orientation (*r* = 0.24) for American participants but not for Turkish participants. See Table 2 for complete bivariate correlations.

### 3.2. Multi-Group Path Analysis

To test the predictor roles of BPNT Autonomy, BPNT Relatedness, BPNT Competence, Ego Orientation, and Task Orientation on Physical Activity between cultures, we ran multi-group path analysis. We controlled for participant sex and age based on previous studies [38,39].

Results from multi-group analysis showed that there was a moderating role of cultural context between predictors (i.e., BPNT Autonomy, BPNT Relatedness, BPNT Competence, Ego Orientation, and Task Orientation) and Physical Activity. Such that, the paths from predictors (i.e., BPNT Autonomy, BPNT Relatedness, BPNT Competence, Ego Orientation, and Task Orientation) to PA was not significant in Turkish cultural context. However, the paths from BPNT Competence (β = 0.751) and Ego Orientation (β = 0.267) to Physical Activity was significant in American cultural context.

See Table 3 for complete results

## 4. Discussion

In sport psychology, motivation is a long-standing fundamental factor in PA sustainability [12,29]. As motivation is a multi-layered notion, it is affected by numerous parameters, including culture as identified in the current study. Although the PA inactivity epidemic spans the globe, cultural differences are rarely examined in sport and exercise psychology contexts with even fewer comparing historically individualistic (United States) and collectivistic (Turkey) countries to enhance our understanding of motivational patterns. Therefore, the present study aimed to draw attention to the influence of culture on PA behavior via SDT and goal orientation. More specifically, the moderating role of culture on goal orientations is studied within the SDT framework to understand the differences of PA in two different cultures. The study revealed that participants from the two nations were significantly different but not overly large on self-construals indicating distinct cultural differences and that Turks were lower in BPNT autonomy in an exercise context and task orientation compared to Americans. Both cultures were similar in BPNT relatedness, BPNT competence, and ego orientation. When bivariate correlations were considered, in Americans, BPNT autonomy, BPNT competence, and both goal orientations were positively associated with PA. For Turks, only BPNT autonomy and relatedness were positively associated with PA. Finally, in multi-group analysis, there was a moderating role of culture for the association between predictors and PA. For Americans, culture moderated the relations between both BPNT competence and ego orientation and PA. For Turks, no associations emerged.

### 4.1. Cultural Differences in Nations

When it comes to self-construals, as opposed to basic needs in exercise, the current study showed that, as expected, Turks were higher in relatedness while Americans were higher in autonomy. It should be noted that, while significantly different, both cultures were at least moderate in each construct. Although cultures are generally classified as either interdependent and high in relatedness or independent and high in autonomy [10], the authors were not surprised that both constructs emerged in line with the notion of autonomy and relatedness coexisting rather than competing [15]. After these differences were noted between participants in Turkey and the United States, nationality was treated as a proxy for culture.

### 4.2. Culture and BPNT

As self-construals showed that differences in cultures existed in the sample, BPNT in exercise could then be evaluated in each culture. It was hypothesized that participants from the United States and Turkey would differ on BPNT autonomy and relatedness in PA such that Turks would have higher levels of relatedness in PA settings, while Americans would have higher levels of autonomy in PA settings. Both groups were expected to have similar levels in need for competence in PA settings. These hypotheses were partially confirmed in that there was a significant difference between Turks and Americans in terms of autonomy in PA. This was an expected finding as autonomy is an important component of individualistic countries such as the United States [40]. However, although Americans had higher levels of BPNT autonomy, there was neither a significant difference in their correlations between BPNT autonomy and PA, nor was there a moderation of the construct by culture. Previous studies explained an autonomy-supportive climate does foster self-determined motivation [41] and based on the results of this study the cultural difference, in PA context, do not contribute further to this relationship.

For BPNT relatedness, in contrast to expectations, the groups did not differ. Although, in bivariate models, BPNT relatedness was positively related to PA among Turks, the construct’s relation to PA was not moderated by culture. A potential reason for not finding a moderation by culture in relatedness might be that college students prefer to participate in PA with their friends [42]. Relatedness items in the BPNES survey were mainly asking about people who you are involved with during exercise (e.g., “My relationships with the people I exercise with are close” and “I feel I have excellent communication with the people I exercise with” [30]. As participants might have responded to these questions according to their relationships with their friends, the results might not generalize to other populations.

With BPNT competence, the last of the three basic psychological needs in exercise, both groups reported similar levels of the construct. As competence is a crucial factor for PA not linked to cultural construals, there were no expectations or observed cultural differences in mean levels. As expected BPNT competence was significantly associated with higher levels of PA in both groups but with no significant difference in relationship strength. Although the groups did not differ in BPNT competence levels, the moderation of competence on PA was unexpectedly different with only Americans having a positive association. Competence is a crucial factor for BPNT, so it was expected that both groups would function similarly and have similar competence scores. However, with the moderation we can observe a different relationship with PA, and thus, it is plausible that people from individualistic cultures such as Americans might have similar levels of competence but a greater need to display and demonstrate it to others [43,44]. Future studies could examine the differences between the actual competence level vs. the need to display.

### 4.3. Culture and Goal Orientation

For goal orientation, as ego orientation was associated with independent cultures [12]. Turks were expected to be more task-oriented and less ego-oriented in their PA participation in comparison to Americans. Although there were no group differences in the level of ego orientation, the construct had a relationship with culture. Not only did Americans have a positive association with ego orientation and PA, but the construct was also moderated by culture. Whereas in Turks, the two variables were essentially unrelated. Therefore, besides focusing on BPNT competence, interventions raising ego orientation and focusing on outcompeting could increase PA in Americans. These relationships are aligned with aforementioned the need of competence display. As ego orientation refers to outperforming your counterpart [45], this might be considered a further sign of competence display for Americans. In addition, PA was the main context for this study which may evoke less need for ego-orientation. Conducting a research in a sport context in which competition and outperforming is required, then ego-orientation might have been more salient [45].

As anticipated, there was a significant difference between Turks and Americans in terms of task orientation. However, the direction of the relationship was counter to what was expected with Americans being higher in the construct compared to Turks and also only Americans showing a relationship between task orientation and PA. Despite these differences, the relationship between task orientation and PA was not moderated by culture. Considering the sample consisted of college students, the majority of the participants might think that they already know the foundations of exercise [46,47]. Their expectations from PA might be more targeted towards enhancing physical appearance, attractiveness and/or having fun and socializing with friends allowing less room for personal development [47,48,49].

### 4.4. Culture and PA Levels

Finally, aside from goal orientation and BPNT, PA levels differed between the two groups with Turks reporting being less physically active than Americans. A potential explanation can be logistics. There are more recreational options in the United States such as walking paths, exercise facilities, and recreational areas within the campus to be physically active compared to Turkey [48]. These aspects of the built environment may encourage university students in the United States to be more physically active.

### 4.5. Limitations and Future Research

Although this cross-cultural study adds to the extremely small literature on culture and exercise, it is important to acknowledge the limitations of this study. This is a cross-sectional study with a limited sample size. Based on the cross-sectional aspect, causation cannot be inferred from the data. Future longitudinal randomized-control studies and interventions targeting autonomy, competence, and ego orientation could assist with this issue. As for the limited the sample size, although the sample was sufficient for detecting effects via power analysis [36,37], the sample is small when representing two entire cultures. Furthermore, the study considers nationality as a proxy for culture. To more fully address this limitation and verify the findings, future research should incorporate participants from multiple representative individualistic and collectivist cultures [11,14]. However, considering the limited number of cross-cultural studies [11,12,17,24,25], drawing attention to cultural factors and initiating further studies is crucial to tackle the global PA epidemic.

Additionally, possible measurement issues may have influenced the self-report results. As the language of education was English, the questionnaires were not translated for Turkish students. However, the potential language barrier posits as a limitation. Although the validity and reliability of items were acceptable, the task and ego orientation in the sports questionnaire (TEOSQ) was not specifically designed for PA purposes. The questionnaire was developed for investigating sport participation, and it was adjusted for this study. Changes involved simply replacing the word “sport” with “physical activity”. Furthermore, as the nature of PA is not competitive; PA mainly involves fun, learning and improving skills. This might have been the main reason for the similarities in scores between the two groups. It would be interesting to look at the same constructs in cross-cultural settings among collegiate athletes or in another sport setting that is competitive rather than a PA setting.

Finally, the measurement of PA is a challenging task, and this study’s findings relied on self-report solely [31]. Participants may have overlooked lower intensity activities such as walking, grocery shopping, or gardening that are ingrained in their daily activity but are more difficult to recall over a 7-day time frame. Secondly, the GLTEQ measures the frequency of PA performed for periods of 15 min or more during a week. All activities that are less than 15 min are not counted. Those shorter-length activities, especially if they are a part of daily routines, might make a significant difference when added up on a weekly basis. Additionally, accurate reporting of PA intensity might be puzzling for some participants as the perception of PA intensity may not match the intensity of a given PA described in the questionnaire. Finally, recall bias is another limitation. Therefore, providing additional self-report data with diary entries or using electronic activity monitoring could improve the measurement sensitivity for PA in future studies.

Additionally, there may be some limitations for the sample in both gender and culture. The sample is over 60% female. Gender was related to PA for Americans with males exercising more and was moderated by culture in the Turkish sample in the same direction. Turkey is a Muslim culture in which females may have more restrictions based on clothing and setting in PA [50]. Therefore, supporting a climate that foster PA participation in females may be needed in such cultures.

When it comes to culture, the limitations the sample yields would likely have resulted in an underestimation of the differences between variables in this study. This statement stems from two reasons. First, the American sample may be less independent and the Turkish sample less interdependent than the national norms, a possible artifact arising from the cities where data collection occurred. The American sample was collected from the University of North Carolina at Greensboro located in North Carolina’s third largest city with a population of 750,000. In contrast, students from Turkey came from Istanbul, Turkey’s largest city with a population of 20 million. The results may have been even more pronounced if the American students came from a big city in the United States such as New York or Los Angeles or if the Turkish students had been recruited from a smaller city. Another potential limitation lies in the nature of the universities. UNCG is a public university whereas Koc University is a private institution. The socioeconomic status might create discrepancies between the two groups of students.

Future research could investigate cultural differences in a comparison between students from independent and interdependent cultures studying at the same university. In this way, groups would have similar opportunities with similar avenues, under the same educational system. Thus, if recreational facilities and opportunities have a significant impact on PA, these environmental confounds would be controlled and the only difference between students would presumably be their culture.

## 5. Conclusions

In conclusion, the PA inactivity epidemic and its ramifications affect the global population, and thus, strategies to increase PA to recommended levels need to account for cultural differences. This study not only reveals differences in levels of BPNT exercise constructs, and goal orientation, but more importantly studies how these concepts are linked to PA behavior in two cultures. In Turks, the factor most closely linked to PA behavior was BPNT autonomy, whereas, for Americans BPNT competence and ego orientation had a stronger relationship. Thus, as cultural differences exist, a culturally tailored approach to PA interventions rather than a one size fits all framework is crucial in battling this issue.

## Figures and Tables

**Table 1:** Descriptive Statistics and Differences between Turks and Americans in terms of self-construals, basic psychological needs in exercise, goal orientation, and Physical Activity.

Variable (Range)	Turkish (n = 92)	American (n = 76)
	M (SD)	M (SD)
Physical Activity	29.05 (20.14) ***	46.74 (24.37) ***
Self-Construal		
Autonomy (1–5)	3.43 (0.68) **	3.70 (0.58) **
Relatedness (1–5)	4.20 (0.66) ***	3.79 (0.82) ***
Basic Psychological Needs in Exercise		
Autonomy (1–5)	3.51 (0.80) *	3.78 (0.80) *
Relatedness (1–5)	3.64 (0.90)	3.52 (0.95)
Competence (1–5)	3.37 (0.86)	3.12 (0.90)
Goal Orientation		
Ego Orientation (1–5)	3.28 (0.83)	3.12 (0.90)
Task Orientation (1–5)	4.02 (0.61) ***	4.33 (0.46) ***

Note. * *p* < 0.05. ** *p* < 0.01. *** *p* < 0.001.

**Table 2:** Correlations among study variables for the U.S. and Turkey samples.

Variable	1	2	3	4	5	6	7	8	9	10
1. Physical Activity	-	0.006	−0.18	0.31 **	0.32 **	0.19	−0.03	0.08	−0.05	−0.18
2. SC Autonomy	0.06	-	−0.39 **	−0.12	−0.16	−0.25 *	0.16	−0.10	0.26 *	−0.21 *
3. SC Relatedness	−0.16	−0.58 **	-	0.14	0.21 *	0.34 **	−0.08	0.15	−0.27 **	0.44 **
4. BPNT Autonomy	0.23 *	−0.12	0.27 *	-	0.76 **	0.56 **	0.23 **	0.24 *	−0.23 *	0.02
5. BPNT Relatedness	0.15	−0.13	0.26 *	0.71 **	-	0.48 **	0.14	0.11	−0.21 *	0.13
6. BPNT Competence	0.51 **	−0.31 **	0.53 **	0.51 **	0.53 **	-	0.18	0.17	−0.26 *	0.13
7. Ego Orientation	0.23 *	−0.06	−0.23 *	0.06	0.003	−0.05	-	0.10	−0.23 *	−0.03
8. Task Orientation	0.24 *	0.02	0.21	0.13	0.19	0.26 *	−0.07	-	−0.14	0.07
9. Age	0.07	0.03	0.03	−0.06	0.06	0.04	−0.21	0.04	-	−0.13
10. Sex (1 = F, 0 = M)	−0.23 *	−0.17	0.16	−0.07	−0.29 *	−0.04	−0.16	−0.15	−0.11	-

Note. Below the diagonal shows correlations for the U.S.; above the diagonal shows correlations for Turkey. * *p* < 0.05. ** *p* < 0.01.

**Table 3:** Results from multi-group analysis: Moderating role of cultural context.

Predictor	American Sample			Turkish Sample		
	Unstandardized Estimate (SE)	Standardized Estimate (SE)	95% Confidence Interval	Unstandardized Estimate (SE)	Standardized Estimate (SE)	95% Confidence Interval
BPNT Autonomy	−8.081 (4.012)	−0.264 (0.141)	−0.568/0.000	2.646 (4.882)	0.105 (0.184)	−0.259/0.457
BPNT Relatedness	−4.010 (2.991)	−0.153 (0.118)	−0.391/0.067	1.160 (2.291)	0.052 (0.101)	−0.142/0.265
BPNT Competence	20.715 (3.223) ***	0.751 (0.130)	0.487/1.004	6.028 (3.810)	0.256 (0.156)	−0.050/0.581
Ego Orientation	7.212 (2.425) **	0.267 (0.088)	0.094/0.428	−2.931 (3.137)	−0.120 (0.125)	−0.362/0.144
Task Orientation	10.073 (5.553)	0.191 (0.102)	−0.012/0.382	1.548 (3.726)	0.047 (0.080)	−0.094/0.222
Age	0.323 (0.520)	0.064 (0.094)	−0.134/0.237	−0.086 (0.682)	−0.015 (0.161)	−0.349/0.271
Sex (1 = F, 0 = M)	2.032 (5.238)	0.041 (0.105)	−0.165/0.250	−9.968 (4.723) *	−0.233 (0.095)	−0.418/−0.049
*R^2^*	0.40			0.17		

Note. * *p* < 0.05; ** *p* < 0.01; ***p < 0.001. Bootstrapping analysis were run with 2000 replications.
